# Distress in patients with end-stage renal disease: Staff perceptions of barriers to the identification of mild-moderate distress and the provision of emotional support

**DOI:** 10.1371/journal.pone.0225269

**Published:** 2019-11-21

**Authors:** Gill Combes, Sarah Damery, Kim Sein, Kerry Allen, Johann Nicholas, Jyoti Baharani

**Affiliations:** 1 Institute of Applied Health Research, University of Birmingham, Edgbaston, Birmingham, United Kingdom; 2 Health Services Management Centre, University of Birmingham, Edgbaston, Birmingham, United Kingdom; 3 New Cross Hospital, Royal Wolverhampton NHS Trust, Wolverhampton, United Kingdom; 4 Birmingham Heartlands Hospital, University Hospitals Birmingham NHS Trust, Birmingham, United Kingdom; The MetroHealth System and Case Western Reserve University, UNITED STATES

## Abstract

**Objectives:**

To explore staff perceptions of barriers to the identification of mild to moderate distress and the provision of emotional support in patients with end-stage renal disease.

**Methods:**

Qualitative semi-structured interviews with staff in two hospitals (n = 31), with data analysed using a hybrid approach combining thematic analysis with aspects of grounded theory.

**Results:**

Staff appeared very aware that many patients with end-stage renal disease experience distress, and most thought distressed patients should be helped as part of routine care. However, practice was variable and looking for and addressing distress was not embedded in care pathways. Interviews identified six themes: i) staff perceptions about how distress is manifested and what causes distress were variable; ii) staff perceptions of patients could lead to distress being overlooked because patients were thought to hide their distress whilst some groups were assumed to be more prone to distress than others; iii) role perceptions varied, with many staff believing it to be their role but not feeling comfortable with it, with doctors being particularly ambivalent; iv) fears held back some staff, who were concerned about what might happen when talking about distress, or who found the emotional load for themselves to be too high; v) staff felt they lacked skills, confidence and training, vi) capacity to respond may be limited, as staff perceive there to be insufficient time, with little or no specialist support services to refer patients to.

**Conclusions:**

Staff perceived significant barriers in identifying and responding to patient distress. Barriers related to skills and knowledge could be addressed through training, with training ideally targeted at staff with positive attitudes, but who currently lack skills and confidence. Barriers related to role perceptions would be harder to address. The study is relevant internationally as part of improving long-term condition pathways.

## Introduction

Treatments for patients with end-stage renal disease (ESRD) are life-sustaining rather than curative, and exert a high physical and mental toll on patients. Emotional and psychological stressors relate to acceptance of diagnosis, disease progression, making treatment choices, coping with treatment regimes, and wider impacts on employment, relationships and lifestyle [1–4]. Estimates of anxiety and depression rates in patients with ESRD range from 20–30% [5,6], and are broadly similar to rates for other long-term conditions such as cancer [7,8], diabetes [9] and chronic obstructive pulmonary disease [10], but far exceed rates in the general population of 7–9% [11–12]. Untreated anxiety and depression in patients with ESRD are associated with decreased health-related quality of life and higher symptom burden [13,14], increased healthcare use, poor diet or medication adherence [15–17], higher risk of withdrawal from dialysis [18–20], and a higher mortality risk than for other chronic conditions [21,22].

For over 10 years, UK policy has highlighted the importance of integrating emotional and psychological health into long-term condition care pathways [23]. This is reflected in renal disease guidelines [24–26], whilst more recent policy emphasises parity between mental and physical health [27,28]. Although support for emotional difficulties is regarded by patients with ESRD as an essential component of effective healthcare [29], support is generally targeted at patients requiring psychiatric or psychological intervention. Lower level needs (‘distress’) in renal patients have tended to go unrecognised and untreated [30,31]. Definitions of distress originate in the oncology field, where distress “extends along a continuum ranging from common normal feelings of vulnerability, sadness and fears, to problems that can become disabling such as depression, anxiety, panic and social isolation” [32]. It has been argued that distress can have similar impacts to clinical depression [33].

There is a lack of evidence about the support that patients with ESRD need and when they need it. Although progression from distress to more severe psychological difficulties is not inevitable, timely identification of distress and provision of support may help to ameliorate the impacts on patients. Renal staff may be particularly well placed to provide emotional and psychological support, as they may develop close relationships with patients over time. However, a few small-scale studies suggest that renal staff find it hard to recognise distress [20,22,34], and evidence from oncology studies suggests that patients tend not to spontaneously express emotional concerns in clinics [8,35], or that they do so indirectly through verbal and non-verbal cues [36]. Furthermore, during time-limited consultations, doctors are reluctant to raise emotional issues, focusing instead on clinical/medical issues [37,38].

Thus, despite evidence that there may be unmet needs related to distress among renal patients, and that it can have significant impacts on patients’ lives, little is known about how staff identify and respond to patient distress, and the barriers they may face. This evidence gap was addressed as part of a larger mixed methods study with patients and staff [39]. In this paper, we report findings from a qualitative study with renal staff which aimed to identify and explore the barriers staff face in identifying and responding to patients with ESRD with mild to moderate distress. The study was expected to be relevant to staff beyond renal services, by informing improvements in patient care across other long-term conditions, given the association between distress and long-term conditions. It was also expected that the study would help build the profile of this issue internationally and would be relevant to low-middle income countries where prevalence rates for long-term conditions continue to rise [40].

## Materials and methods

The study was approved by the NRES West Midlands Coventry and Warwickshire Research Ethics Committee (ref: 15/WM/0288) and the Health Research Authority (IRAS project ID 184996). Approval was also obtained from the research governance office of each site.

Semi-structured, in-depth qualitative interviews were undertaken with renal staff from two hospitals with diverse patient populations located in urban areas in the West Midlands, England. Table 1 summarises the key characteristics of the two hospitals in 2016 [41].

**Table 1:** Hospital site characteristics.

	Site 1	Site 2
Catchment population	670,000	740,000
% BME^a^ population	32.3%	39.1%
Median age RRT patients	60.6 years	65.1 years
No. patients on dialysis	384	483
No. transplant patients	185	171
No. acute beds	30	34

^a^BME: black and minority ethnic

Potential interviewees were identified from their responses to a staff survey carried out as part of the larger study. Staff were then purposively selected for interview in order to achieve maximum variation by four role categories in each site (consultants; dialysis unit nurses; other renal nurses; and allied professional renal staff including dieticians, psychologists and social workers). Participation in principle was confirmed by telephone with a consent form and Participant Information Sheet sent by email, followed by a phone call one week later to confirm participation and arrange an interview time. Telephone or face-to-face interviews at staff members’ workplaces were undertaken between April and December 2016 by two female qualitative academic researchers (FT, EK) who were qualified to Masters level and employed by the University of Birmingham. Written consent was obtained from all participants. Four staff had participated in previous research undertaken by the team, but most had not and did not know the interviewers. The interviewers had no personal experience of, or particular personal interest in, the research topic.

One-to-one interviews were selected as the best method for encouraging professional staff to express views as freely as possible on a topic which was potentially sensitive, as the literature suggested national guidance on emotional support was not being fully implemented. Face-to-face interviews lasted 30–60 minutes, involved one interviewer and one interviewee at a time, and took place in a private room where interviewees could not be overheard. Brief field notes were made, as appropriate, after each interview. The topic guide was informed by the literature, renal patients who were advisors to the project, and renal clinicians and academics on the project’s Advisory Group. A small number of question prompts were designed to elicit views about: how distressed patients were identified and supported; factors that helped and hindered this; what support was needed by which patients and when; whose role it was to identify and respond to patient distress; how skilled and confident staff felt about this; what needed to change and how this could be done (S1 Table). Distress was defined at the start of the interview as: patients experiencing higher stress, poorer emotional adjustment and worse quality of life compared with the general population, arising from diagnosis and/or treatment, and excludes any diagnosed psychological/psychiatric conditions.

Data saturation was reached after 31 interviews had been completed. There were no participant withdrawals. Interviews were audio-recorded and professionally transcribed verbatim, with transcripts proof-read against recordings to check for accuracy. Interviewees did not have the opportunity to check transcripts. A hybrid approach to analysis was used, combining aspects of grounded theory [42] and thematic analysis [43]. Interview transcripts were initially analysed inductively, since there was no relevant theory or literature to draw on, using relevant aspects of grounded theory (open coding and constant comparison analysis). Two researchers (FT, EK) coded the first five transcripts independently, and following comparison and discussion, developed an initial coding framework. This was used by KS to code the remaining transcripts using NVivo software. During coding, regular discussion of ordinate and subordinate nodes led to refinement of the detailed coding structure.

Where data did not fit existing themes, new codes were developed or existing ones revised. There were a small number of differences between researchers in how they coded the data early on in the coding. These were resolved by the team revisiting the relevant sections of the transcripts and discussing the conflicting codes until there was unanimous agreement about the most appropriate codes to use. After coding had been completed, a random selection of 10% transcripts was coded independently by GC to check for consistency, with minor amendments subsequently made to the coding. Thematic analysis was then used to compare and cluster codes in order to identify emerging themes and sub-themes, which were discussed by the research team and refined further through an iterative process of additional analysis and discussion. Triangulation and synthesis across sites to identify overall study findings was followed by discussion of findings with clinical staff at site-specific feedback meetings, leading to further refinement of the findings. Research team meetings were used to discuss reflexivity and consider how to minimise the influence of individual researchers on the findings.

## Results

All staff who were approached for interview agreed to take part. Thirty-one interviews were conducted, 16 at site 1 and 15 at site 2 (Table 2). Seven staff were interviewed face-to-face at the renal unit, and 24 by telephone. Participants were spread fairly evenly between the four role categories of consultants (n = 7), dialysis unit nurses (n = 10), other renal nurses (n = 8), and allied professional renal staff (n = 6) and were evenly balanced by role category across sites (S2 Table).

**Table 2:** Staff interviews by role and site.

Staff roles	Site 1	Site 2	Total
Renal consultant lead	1	1	2
Renal consultant	3	2	5
**Consultant total**	**4**	**3**	**7**
Haemodialysis unit nurse manager	0	1	1
Haemodialysis nurse	5	4	9
Renal ward nurse	0	1	1
Specialist renal nurse (pre-dialysis; peritoneal dialysis; home haemodialysis)	3	3	6
Renal research nurse	1	0	1
**Nurse total**	**9**	**9**	**18**
Renal dietician	2	1	3
Renal social worker	0	1	1
Renal occupational therapist	0	1	1
Welfare rights officer	1	0	1
**Allied professional staff total**	**3**	**3**	**6**
**TOTAL**	**16**	**15**	**31**

Analysis identified multiple barriers which prevent or reduce the capacity and capability of staff to identify and respond to patients’ distress. Barriers fell into two broad groups: those related directly to perceptions of patients and distress; and those related to staff roles, skills, confidence and capacity. The themes and sub-themes are summarised in Table 3.

**Table 3:** Interview themes and sub-themes.

	Themes	Sub-themes
Staff perceptions about distress in ESRD patients	Perceptions related to distress	How distress is manifested
		Change triggers distress
	Perceptions related to patients	It’s the patient’s responsibility to tell staff when they are distressed
		Patients may hide their distress
		Some groups more prone to distress than others
Staff roles, skills and capacity	Staff role perceptions	Its everyone’s role, but it’s not my role
		Ambivalence about the role of doctors
		Haemodialysis unit staff prioritise technical care
	Fears hold back some staff	What might happen when we talk about distress
		Emotional load
	Staff lack skills, confidence and training	Knowledge and skills training
		Scepticism about the value of training
	Limited capacity to respond	Lack of time
		Variable access to specialist services

### Staff perceptions about distress in ESRD patients

Most staff considered the prevalence of distress to be high, and an inevitable consequence of ESRD. They wanted help to be provided to distressed patients, and for this to be integral to routine patient care. However, they knew from experience that patients who were not supported well could disengage from the service and potentially have worse medical and psychological outcomes. In contrast, patients who were well supported emotionally were seen as more likely to engage with staff and with their own care, which could have long-term health benefits. Despite this, neither study site had routine processes for identifying patient distress, and it was left up to individual staff to do what they felt was appropriate, if anything.

Although staff were aware that many patients may experience distress, they talked at length about finding it hard to identify. The analysis identified a number of underlying perceptions related to how staff view distress, and how they view ESRD patients. These are now explored in detail.

#### Perceptions related to distress

How distress is manifested. Some staff believed that distress is usually manifested through changes in patients’ body language or behaviour. They described relying on their own observations of patients to detect distress. If patients appeared unusually upset, moody or angry, this would trigger a conversation about how they were feeling:

*“It’s looking out for the signs*, *it could be anything; somebody quieter than normal*. *It could even be somebody more chattier than normal*, *noticing something different… it’s very difficult to put your finger on it* [recognising distress].*”*

A4, Welfare rights officer

A sudden change in patient compliance with treatment, diet or fluid intake could likewise trigger looking for distress as a possible cause. Other staff talked about more subtle cues which may alert them to the possibility of distress, and used words such as “intuition”, “clinical experience” and “sixth sense” to describe how they might know a patient is distressed.

The interplay between physical and psychological health was raised as problematic by some staff. They expressed a belief that psychological and emotional problems can masquerade as physical problems such as pain, or that some emotional problems can in fact have a physical root related to ESRD. This could lead to confusion about what were the most appropriate actions to take to help patients.

Change triggers distress. Many staff believed that patients were most likely to be distressed at times of change, in terms of their treatment or in their lives more generally. Three points on the pathway were identified as most likely to trigger distress: on reaching the end stage of their renal disease; during pre-dialysis preparation; and when treatment first starts:

*“OK*, *it’s either very early on*, *where it’s almost a shock situation when somebody’s first been diagnosed with* [end-stage renal] *failure*, *pre-dialysis almost*. *And I think once they start dialysing as well*, *because that’s a very*, *it’s very unfamiliar… almost a settling-in phase*.*”*

B2, Haemodialysis nurse

For other staff, emotional support was thought to be most likely to be needed when a treatment becomes difficult or needs to change:

*“… they’ve been on peritoneal dialysis and they’re moving to haemodialysis*, *then that’s a big change and they might need to be support*[ed] *there*. *Because it can be quite a confusing time*, *it’s a big change and a very difficult thing to do*. *So I think at each of the points where things change*, *where perhaps it would be good for people to be offered*, *sort of*, *a more formal opportunity to talk through what their issues are and any concerns they have*.*”*

A9, Renal dietician

Staff highlighted that although points of change in patients’ treatment might be *more* likely to lead to distress, ironically, the need to deal with these changes clinically can often lead to there being much *less* time available than usual for providing support:

*“And it’s not because we don’t want to, we know in the back of the mind, the more challenging the patient medically is, proportionately the emotional side of things also will be quite high. But the time which we have will be largely focused on the medical side of things and then they miss out on the emotional side of it”*.

A7, Consultant

The move to a new treatment also usually involves a change in the staff who care for patients. Patients must then relate to new staff who don’t know them and who may be less likely to notice their distress. Although some staff found it useful to think about change as a trigger for distress they acknowledged that some patients may try to ‘get through’ changes by deferring their distress, which only surfaces later on.

#### Perceptions related to patients

It’s the patient’s responsibility to tell staff when they are distressed. In contrast to the views explored in the previous section, where staff described using their experience and skills to judge when patients were distressed, there were other staff who believed that the absence of obvious outward signs meant patients were not distressed. If a patient appeared to be happy on the surface, they assumed nothing was wrong, and their default position was therefore not to ask patients how they were feeling. They did not see it as their role to probe or actively look for distress, assuming that distressed patients would raise concerns themselves or initiate a conversation about how they were feeling:

*“I would like to think that if they* [the patients] *were feeling like that* [distressed] *they would say…*.*”*

A2, Haemodialysis nurse

However, others had found from experience that patients may not volunteer information about how they are feeling and may be waiting to be asked by staff:

*“Sometimes people are just looking for somebody to ask them the question and they will tell you ‘actually I feel pretty lousy’ or ‘actually things have been awful with my fistula this month and my sick pay has stopped and I can’t pay the bills and I just feel dreadful and I really don’t know how I’m going to get out of this one’*.*”*

B11, Renal social worker

Patients may hide their distress. One doctor eloquently summarised some of the reasons why patients may not show their distress to staff:

*“So there’ll be some that definitely want their privacy*, *there’ll be some people that won’t think us effective*, *there are others that are embarrassed and there are others that don’t think it’s the right place to mention* [distress], *and the others that don’t know how to articulate it*.*”*

A17, Consultant

In addition, staff thought that some patients may want to avoid being labelled as not coping, or may see distress as taboo, equating it with mental health problems:

*“And just the admission that you need support, it’s just for all of us isn’t it, we all like to be self-sufficient…they will access support from our benefits advisor when they have financial difficulties, quite regularly, because the disease has caused that, rather than them being the problem… they’ll shout when they’re short of money, but not when they’re crumbling psychologically*.*”*

A5, Specialist renal nurse

Some staff also thought that patients’ perceptions of how staff work could mitigate against the identification of distress: patients see staff as increasingly pressed for time and conclude there is no time to ask about how they feel. Consequently, patients want to avoid adding to pressures on staff they know well and may like. In addition, patients may feel they are ‘letting down’ staff if they admit to feeling low or not coping, with the desire to be a ‘good patient’ applying particularly to interactions with doctors:

*“I think there’s a tendency, it depends a little bit on the doctor, but I think patients are seeing medical staff less frequently and they may put on a bit of a show for a doctor*.*”*

B10, Consultant

Thus it appeared that the long-term nature of clinical relationships with renal patients who know staff well and see them over many years, may inadvertently work against the identification of distress.

Some groups more prone to distress than others. Views varied about whether some patients were more prone to distress than others, with discussion of potentially unhelpful stereotypes about: men, who were perceived as not liking to talk about emotions; younger patients, who were perceived as needing more support; and older people, who were perceived as stoical and less likely to want support. However, staff also emphasised individual differences and the importance of understanding each patient’s distress reactions and circumstances:

*“Some of the people who come in won’t have family support at home. So I think if we can provide a talking point for them when they come in, I think they’re more likely to come in and have the dialysis*.*”*

B12, Specialist renal nurse

Black and ethnic minority patients were simultaneously viewed as likely to experience more distress than other ethnic groups, but less likely to need support. Some staff assumed family and community networks were helpful, so that patients were less likely to need emotional support:

*“Usually, the Asian community they sort of look after their own groups because I think they’re very community based…and everybody supports each other*.*”*

B15, Haemodialysis nurse

For others, family support was seen as double-edged, and could potentially contribute to patients’ distress:

*“Once they become a kidney failure patient and start dialysis, then a lot of their autonomy is taken away by the family and a lot of decision making process will then automatically shift to the family rather than the patient….from the outside it looks like they’ve got more family support, but actually that’s not really helping them. Yes it does help them in physical looking after them, but I think the emotional side of things, they are more vulnerable than the Caucasians I think*.*”*

A7, Consultant

Several staff observed that Asian men were unlikely to talk about their feelings, whilst Asian women were seen as overtly expressing emotional need, although sometimes this was demonstrated in different ways from other ethnic groups:

*“I’m just using the stereotype of an Asian lady that she will have the complaint of pain all over, but maybe that’s the way she can emote*.*”*

B5, Consultant

### Staff roles, skills and capacity

#### Staff role perceptions

It’s everyone’s role, but it’s not my role. A prevailing view in the interviews was that identifying and responding to patient distress is *everyone’s* role in the renal unit, including non-clinical staff:

*“We all have that role*. *We all have that responsibility to do that*. *Whether it’s a domestic*, *whether it’s a HCA* [Healthcare Assistant], *whether its band six*, *band five*, *doctor*, *we all have that responsibility*.*”*

B15, Haemodialysis nurse

However, there was a recognition that this may not happen in practice, with some staff thinking that dealing with distress was optional and depended on whether or not individuals have a particular interest in or inclination to deal with it:

*“Some people would see it like ‘well, it’s not my job’. You get that sort of attitude. And then other people like myself you know I’m as soft as anything…. some people will be more interested in that side of things than other people*.*”*

A6, Haemodialysis nurse

Staff identified four factors which may determine whose role it is: personal interest, skills, seniority and opportunity. Seniority was seen as both a pro and a con, with some nurses thinking that doctors should talk about emotional issues, due to the serious nature of the topic, whilst some doctors thought that their seniority might inhibit patients:

*“Even though I don’t think I’m intimidating*, [the patients] *might be intimidated by coming to see a consultant*. *Some people might not tell me things that they’d be quite prepared to tell the cleaner or a nurse or a physio*.*”*

B4, Consultant

During the exploration of whose role it is, an interesting dichotomy emerged. Doctors frequently saw nurses as best placed to deal with distress because they see patients more frequently and were perceived as having better emotional skills than doctors. However, nurses either saw doctors as better placed to deal with distress due to their seniority, or other nurses who had more frequent patient contact. Overall, each staff group was quick to identify another staff group as better placed than themselves to deal with patient distress, and it is likely that this results in some patients’ distress not being dealt with.

Ambivalence about the role of doctors. Most of the doctors who were interviewed thought that dealing with patient distress requires a different approach compared with medical care, and that this can lead to some challenges and tensions for them. For some, dealing with patients’ distress was seen as potentially threatening to their objectivity and detachment, which were considered essential for fulfilling their ‘medical’ role:

“…if I don’t have a degree of emotional detachment, I can’t do what I need to do for them…”

A8, Consultant

The implication was that doctors may find it hard to remain detached when talking about distress, and may get drawn into issues they cannot handle, leading to unwanted personal pressures:

*“…you see an awful lot of problems in medicine and it is very difficult to manage the emotional pressure of that if you get too involved. So there’s a personal issue as well in terms of stepping back a little bit so that you don’t get too sort of personally upset by things*.*”*

A8, Consultant

For some, the challenging part of dealing with distress is that solutions may be harder to find compared with medical issues:

*“Medical problems*, *this is a problem*, *this is a solution…you can sort it*. *But when it comes to the emotional side of things or depressive side of things*, *then it’s* [a] *challenge*.*”*

A7, Consultant

This appeared to lead to feelings of discomfort or helplessness:

*“I mean why open up a can of worms that you can’t actually address. If the patient says to you ‘I’m really depressed’… can you do anything about it? You can talk to them but its empty words really, isn’t it? …On the other hand if you’ve got somebody with anaemia you can deal with it…I can give them an endoscopy, I can stop their aspirin. There’s so many things that I can do and I’m comfortable doing those things*.*”*

B5, Consultant

Overall, there appeared to be considerable ambivalence about the role of doctors in dealing with patient distress, and no consensus among the doctors about what that role should be.

Haemodialysis unit staff prioritise technical care. Staff working in haemodialysis units were highlighted by many interviewees as the group of staff least likely to see patients’ emotional wellbeing as part of their role. This group of staff was seen by many as overly focused on the technical aspects of dialysis:

*“But I just find like some nurses, caring and good as they are, they just see it as, do the dialysis and then get, you know, carry on to the next group, and it’s all more like a production line rather than actually, you know, a caring role*.*”*

B3, Haemodialysis nurse

Despite patients in haemodialysis units having far more contact with staff than other groups of patients, it seems that they are probably the least likely to be cared for emotionally by staff. There was a view that this is increasingly the case, as more and more patients need haemodialysis:

*“Well some* [dialysis] *units are so under pressure*, *they are just processing*, *it’s a dialysis factory*, *you know*, *they’re processing treatments…we’ve got people queuing up for the treatment*, *let’s get them through sort of thing*. *And I think it’s the emotional support and the passion that can get lost very quickly when that’s the case*.*”*

B7, Consultant

#### Fears hold back some staff

Fears about what might happen when we talk about distress. A number of staff had fears related to talking about distress. Some said that although they could identify patient distress, they avoided it because they just did not feel comfortable in talking about feelings, either in general, or with patients:

*“I feel a bit uncomfortable with too much emotion, I’ll be frank*.*”*

B4, Consultant

Others were fearful that patients might not open up to them, and that this would cause awkwardness in the future, whilst other staff appeared to fear uncovering problems they didn’t know how to deal with:

*“I’m just wanting, you know, to move through the consultation quickly without uncovering something that might be a problem… why go there if you don’t have to*.*”*

A8, Consultant

In a similar vein, there was a fear of saying the wrong thing or making things worse rather than better for patients:

*“At the end of the day you could say something really wrong couldn’t you…how do you know you’re saying the right thing? You don’t do you*.*”*

B13, Haemodialysis nurse

Emotional load. Some staff were fearful about the emotional impact on themselves of dealing with patients’ distress:

*“It’s not easy to provide comfort when somebody is very distressed*. *Not everybody can manage that very well*. *We all struggle at times*, *I do*. *And often if you know that person*, *you can feel distressed* [too], *but it’s managing that*.*”*

B9, Renal dietician

Some thought that it was only possible to deal with patients’ emotional problems when everything was going well in their own life. If they had personal problems, they may not have the capacity to deal with the extra emotional load of patients’ distress:

*“…if you are not strong emotionally yourself*, *or if you are going through stuff at home*, *you find that by the time you’ve come to the* [renal] *unit you have nothing to give… So* [the nurses] *have to deal with their* [own] *emotional situation before they can help others*.*”*

A1, Haemodialysis nurse

Some staff seemed conflicted about their role in dealing with patients’ emotional problems, and had difficulty describing how they dealt with them, using stilted speech patterns, lengthy pauses and abrupt changes of sentence structure. They seemed conflicted and this came out in the slightly unclear way they talked about dealing with emotional distress. For some, this conflict seemed to be resolved by actively avoiding any discussion of distress with patients, or assuming that other staff would deal with emotional problems:

*“So those* [emotional problems] *are the things that sometimes it’s better to avoid*. *And then if it’s there*, *we know*, *then we can ask for the nurses to speak to the family or get a psychologist to see* [them]. *And the problem then shifts if we get the psychologist to deal with it*.*”*

A7, Consultant

#### Staff lack skills, confidence and training

Knowledge and skills training. Training emerged as a key issue for staff. None of the interviewees had received any training at all about how to identify and respond to patient distress, although a few had found that some of their learning from mental health or palliative care training was relevant, and some had developed skills over time simply through experience. Staff highlighted both knowledge and skills training as necessary. Many of the interviewees talked about not feeling confident in dealing with patients’ distress because they lacked knowledge about how to identify it and how to support patients:

*“I think it’s got to be training… identifying [distress] and when I need to support the patients. Yeah, because without that, without that background knowledge I feel like, you know, useless*.*”*

A6, Haemodialysis nurse

Other staff focused more on communication skills, wanting to enhance their existing skills specifically in relation to distress. Common suggestions were about how to raise the topic with patients, techniques to get them talking and whether there are things to avoid saying:

*“I always feel like I don’t know what to say, you know when people get that upset*.*”*

A8, Consultant

*“… how to word questions, and how to respond to patients*.*”*

A9, Renal dietician

*“How to approach people, if there’s key words, or picking up on signs. Or if there’s, you know, if there’s no-no’s you shouldn’t do*.*”*

A13, Renal research nurse

Several staff were aware that different consultation styles could make it more or less likely that patients would talk about their feelings. They thought that all staff would therefore benefit from training about this, with an emphasis on ways of developing and showing empathy with patients. Many interviewees also talked about being unsure about when to refer patients to other services and what services exist to help distressed patients. This was particularly the case for referrals to psychology:

*“Just what could be achieved? You know I think we get quite imbibed in the physical and we know what dialysis does and we know what transplant does, but quite what does the psychologist do? You know we’ll send the patient there and hope they come back better and in a better mood perhaps. But I don’t think people are quite aware of what they can achieve*.*”*

A5, Specialist nurse

In one site, the lack of staff training was linked to the lack of a renal psychology service. Staff were aware that in other renal units, psychologists may provide training in the use of specific psychological techniques that staff could use with patients:

*“I mean, there’s a lot of good techniques out there but we don’t have access to them. We don’t have a clinical psychologist attached to the unit, which you know*.*”*

B8, Renal dietician

Overall, it seems likely that staff who avoid patient distress or feel uncomfortable with the topic could potentially overcome some of their fears and feel more confident if they had basic training about the causes of distress, how to talk about it and what can help to alleviate it (Table 4).

**Table 4:** The main training issues identified by staff.

Identification of distress	Which patients are most likely to experience distress and when
	How to spot distress; direct and indirect signs
	Why patients may hide their distress
Responding to patient distress	Understanding our own fears as staff and what holds us back
	What patients want
	Dos and don’ts; what can go wrong
	Practising encounters with different patient scenarios
	Knowledge of what services are available to refer to
	Psychological techniques and how they help
	What psychologists do and when to refer to them
Communication skills	Active listening and empathy
	Basic counselling skills
	How to get patients to open up
	Closing down a conversation about distress
	Different consultation styles and how they help/hinder

Scepticism about whether training will help. Although many staff wanted to see education and training provided, others were sceptical about whether it would help. They tended to debate with themselves whether some staff are inherently better at dealing with patients’ distress, due to experience and personal qualities, and therefore whether education and training can really make a difference:

*“Well I’d like to see more education but, you know, I don’t know if you can teach this kind of thing. I think with that, it’s either experience or education, I don’t know. I think it comes with experience and what you’ve learned from others, whereas other people learn it through education so I don’t know. So it’s a combination of both, education and experience*.*”*

B15, Specialist renal nurse

*“Sometimes you have to have life experiences to be able to relate to someone. And I know you can’t teach life experiences but you can teach counselling and listening. So if people can have the basics of actively listening then that might help*.*”*

A14, Haemodialysis nurse

#### Limited capacity to respond

Time. Lack of time was raised frequently as a barrier, with several dimensions of the issue discussed. Some staff observed that there is now less time to talk with patients, compared with even a few years ago, particularly for ward nurses and dialysis unit nurses:

*“I think probably the immediate thing that comes to me, is time for patients. And I don’t think its rocket science, I don’t think that staff need to deliver something, they just need to have time to talk to patients, maybe just to listen, maybe not intervening in a conventional sense. And I think that’s a component, that’s something that is of value, that’s diminishing or under huge pressure really*.*”*

B10, Consultant

They talked about the external and team pressures to prioritise clinical care, in order to achieve externally required medical outcomes:

*“I want to focus on the medical side first and then deal with that*. *And sometimes I might not have enough time* [for the emotional side].*”*

B1, Haemodialysis nurse

Emotional aspects of care, which are harder to measure, may therefore be left to the end of appointments or left out altogether if time is short. This was particularly the case for some of the doctors:

*“I guess to a certain extent there’s the time factor. There are certain things that I have to achieve in a consultation and it’s very hard to measure the emotional support. There’s a lot of talk about patient experience but that doesn’t always fit with the hard outcomes that we need to achieve*.*”*

A8, Consultant

Concerns were also expressed about the unpredictable nature of asking patients about their emotional wellbeing and the impact this can have on staff time and workload. Time is needed to get patients to open up, but the amount of additional time then needed to discuss feelings and any required actions is unpredictable:

*“If you ask the question ‘how are you?’ you must be prepared to sit and listen to the full answer. That’s important. For example, from that answer it will tell you what to do next. And it’s not the sort of thing you can do when you’re doing something else*.*”*

A10, Renal dietician

Some of the doctors were very focused on time as a major barrier:

*“And how to do it* [talk about emotional problems] *in a short time*? *If we have the principles to solve that in 10/15 minutes*, *that’s fine*. *We have that sort of time*. *But if the discussion—if we have to spend half an hour or a bit longer than that*, *then that’s difficult*.*”*

A7, Consultant

If, as this consultant suggested, staff were confident that talking about emotional problems would only take 15 minutes, they might feel more comfortable having those discussions. Without the knowledge of how to deal with emotional problems in a time-limited way, some staff appeared reluctant to start conversations with patients about their emotional wellbeing.

Variable access to specialist services. Staff commented at length that they do not have adequate access to specialist support services. Renal psychology was seen as the most important specialist service. It seems likely that this was highlighted because one site did not have a renal psychologist to which patients could be referred. However, even in the site with a renal psychologist, waiting times were a problem, whilst staff thought that some patients were reluctant to be referred due to perceived stigma. Staff also expressed frustration about lack of access to community mental health services and social services to help patients manage the wider impacts of ESRD.

Overall, we identified three broad groups of staff, based on their attitudes, beliefs and experience, which we summarise as: Enthusiasts, Equivocators and Avoiders (Table 5). Although not all staff will fit neatly into these groups, they may help when thinking about targeting improvements or interventions. For example, Enthusiasts are unlikely to need training but would benefit from peer support in order to share techniques for dealing with patients’ distress and to avoid burnout. The Equivocators, who are mostly able to identify distress but are not confident in how to respond, would benefit most from training. In contrast, the Avoiders are unlikely to change until the identification and management of patient distress becomes embedded into routine patient care.

**Table 5:** Characteristics of three staff groups related to the identification and management of patient distress.

Enthusiasts	Equivocators	Avoiders
Intrinsic to their role	Important part of role in theory but not practice	Know it’s important to patients but definitely not their role
Skilled and confident	Mixed feelings, unsure about skills and confidence	Not comfortable with emotional talk, lack skills and disposition
Proactive in identifying and managing distress	Not proactive, tend to identify but look to others to manage distress	Avoid both identifying and responding to distress
Critical of colleagues in other two groups	Prefer others to take this role	Others much better placed to take this role
Small group of very experienced staff in a variety of roles	Largest group, mostly nurses and other renal staff	Relatively small group, mostly consultants and a few nurses

The Enthusiasts were a relatively small group, made up mostly of highly experienced specialist nurses plus a small number of consultants. The Equivocators were the largest group, made up of haemodialysis and specialist nurses, and allied professional renal staff. The Avoiders were also a relatively small group, made up mostly of consultants and a small number of haemodialysis nurses.

## Discussion

This study identified multiple barriers for staff in identifying and responding to distress in patients with ESRD, which have been explored in detail. Overall, we found that two fundamental building blocks for clinical practice were not in place: knowledge of the topic and the skills required to address it. Improving staff knowledge about distress and developing staff members’ skills to talk with patients could be addressed through training, and the literature from other clinical specialties, particularly oncology, suggests this is likely to lead to an increase in staff confidence in dealing with distress [44]. Without it, staff have a dilemma. Even if they are able to identify distress, and believe it is an important part of care, most are not confident to help distressed patients. By ignoring distress or referring distressed patients to someone else, the ‘problem’ of distress essentially goes away, but it reinforces staff members’ views that they lack confidence and experience in helping patients with distress.

In the absence of relevant renal literature, we now discuss the findings in relation to the oncology literature, as this speciality is at the forefront of psychosocial care, with three decades of experience and research to draw on. It became apparent that both sites had variable and unstandardized approaches to patient distress, which led to some patient distress not being identified and/or responded to, a finding in common with the oncology literature [36,44]. Most of the barriers we identified are similar to those found in oncology services: staff preference for/prioritising medical care [36,45]; differences between doctors and nurses in role perceptions [46]; difficulties identifying distress unless directly expressed by patients [36,46]; unwillingness to probe for distress [44,47]; not knowing how to handle distressed patients [44,46,47]; fear of getting too involved/saying the wrong thing [45,48]; negative emotional impacts on staff [45,48]; lack of time [45,46,48]; lack of skills/training [45,46], and lack of services for onward referral [44–46]. Interestingly, consultation style was only identified as a barrier by a few staff, although a recent study with oncologists suggested that fewer distress cues are expressed by patients during tightly structured physician-led consultations [36].

Barriers related to role perceptions and fears may be much harder to address. Beliefs about doctor/nurse roles appeared to influence whether or not staff thought that emotional care was part of their own/others’ roles. This was most apparent for the doctors who tended to prioritise medical over emotional care, despite recognising that emotional wellbeing affects physical health. Role perceptions were also particularly significant for staff working in haemodialysis units, who were seen as overly focused on technical and ‘machine matters’, to the detriment of caring, a finding which resonates with the literature on task versus caring for oncology nurses [45].

Having enough time for emotional care is also a crucial issue, and was the most cited staff barrier in a recent oncology systematic review [45]. Staff in our study thought that patients may self-censor in relation to distress if they perceive staff to be too busy, which mirrors findings from a recent breast cancer study [36]. It is hard to argue against staff being increasingly pressed for time in the NHS. However, staff could be more skilled at knowing how to initiate then close down conversations with distressed patients in a time-limited way. Acquiring this skill would allow almost any member of staff to take on the role of identifying distressed patients, whilst the provision of emotional support may take longer and therefore need to be done by others or at another time.

Ironically, the study found that staff working with the biggest group of end-stage renal patients–those on in-centre haemodialysis, having treatment three times a week–were the least likely to see emotional support as part of their role. In theory, seeing patients three times a week provides unique opportunities for staff to build strong relationships with patients, and by getting to know them well it becomes more likely that distress is identified. However, in practice, these opportunities are being missed, with staff focussing more and more on the technical aspects of dialysis, as they feel increasing pressure to get patients treated and sent home with the minimum of delays.

Finally, although this study suggests that staff skills and capacity to identify and respond to patients’ distress could be significantly improved for patient benefit, it is possible that the ongoing therapeutic relationships that staff have with patients per se, may itself help to lessen distress for some patients.

The main limitation of the study was that no unqualified staff were included in the interviews. As healthcare assistants make up a significant part of the workforce, particularly in haemodialysis units, this could have biased findings.

## Conclusions

Despite more than 10 years of UK national policy and guidelines emphasising the importance of integrating emotional and psychological health into care pathways for people with long-term conditions, this study suggests that for renal patients, little progress has been made. Staff in this study knew that patients with ESRD experience distress and most believed it was their role to identify and manage it. However, there was considerable variation between staff in whether and how far this was seen as a routine part of their clinical practice. The identification of multiple barriers goes some way to explaining this observed difference between policy and practice. Some barriers may be relatively easy to address through knowledge and skills training. Others are more fundamental, relating to fears and beliefs about professional roles. These barriers are likely to remain until identifying and responding to patient distress becomes part of the culture, rather than the mission of the enthusiastic few, and is reinforced as the norm at both team and organisational levels. The findings of this study are likely to be relevant to a number of other long-term conditions where levels of distress are known to be high. Many of the staff barriers to supporting distressed patients are also likely to be relevant internationally, including in low-middle income countries where the prevalence of long-term conditions is increasing and where the links between distress and patient outcomes may be even less well recognised than in high income countries.

## Supporting information

S1 Table Interview topic guide. (DOCX)

S2 Table Demographic background of staff interviewees. (DOCX)
