# Supplementary material for: Distress in patients with end-stage renal disease: Staff perceptions of barriers to the identification of mild-moderate distress and the provision of emotional support
Source: PLoS One. 2019 Nov 21;14(11):e0225269. doi: 10.1371/journal.pone.0225269 (PMC6871782; doi:10.1371/journal.pone.0225269)
Supplement: S1 Table — (DOCX) [file pone.0225269.s001.docx]

How are distressed patients identified and supported by the staff members/renal unit/hospital?

What is the role of the individual/renal unit/hospital in identifying and responding to distress?

What factors help or hinder support being provided to distressed patients (prompts: perceived capacity, time available, referral options)

Which patient groups need emotional and psychological support most, at what points in the ESRD pathway and for how long?

What are the components of good emotional and psychological support?

How skilled, confident and trained so staff members feel in identifying and supporting distressed patients?

What needs to change or improve to enable better support for distressed patients (interventions, tools, resources, training)?

How could suggested changes or improvements be facilitated and effectively implemented?
