# Supplementary material for: Distress in patients with end-stage renal disease: Staff perceptions of barriers to the identification of mild-moderate distress and the provision of emotional support
Source: PLoS One. 2019 Nov 21;14(11):e0225269. doi: 10.1371/journal.pone.0225269 (PMC6871782; doi:10.1371/journal.pone.0225269)
Supplement: S2 Table — (DOCX) [file pone.0225269.s002.docx]

|  | **Gender** | **Role** | **Years in current role** | **Years since qualification** | **Patient contact frequency** |
| --- | --- | --- | --- | --- | --- |
| Site A | | | | | |
| A1 | F | Haemodialysis nurse | 10-20 | 1-2 | 3-4 days per week |
| A2 | F | Haemodialysis nurse | 20-30 | 30+ | Daily |
| A3 | F | Specialist renal nurse | 1-2 | 5-10 | Daily |
| A4 | F | Welfare rights officer | 3-5 | 30+_ | Daily |
| A5 | F | Specialist renal nurse | 5-10 | 30+ | Daily |
| A6 | F | Haemodialysis nurse | 2-3 | 3-5 | Daily |
| A7 | M | Consultant | < 0.5 | 10-20 | Most days |
| A8 | M | Consultant | 1-2 | 20-30 | Daily |
| A9 | F | Renal dietician | < 0.5 | 3-5 | 3-4 days per week |
| A10 | F | Renal dietician | 10-20 | 20-30 | Daily |
| A11 | M | Consultant | 10-20 | 20-30 | Most days |
| A12 | F | Specialist renal nurse | 10-20 | 20-30 | Daily |
| A13 | F | Renal research nurse | 0.5-1 | 5-10 | Most days |
| A14 | F | Haemodialysis nurse | 10-20 | 20-30 | Daily |
| A15 | F | Haemodialysis nurse | 10-20 | 20-30 | Daily |
| A16 | F | Haemodialysis nurse | 20-30 | 30+ | Daily |
| A17 | M | Consultant | 10-20 | 30+ | Most days |
| Site B | | | | | |
| B1 | F | Haemodialysis nurse | 10-20 | 20-30 | 3-4 days per week |
| B2 | F | Haemodialysis nurse | 10-20 | 20-30 | 3-4 days per week |
| B3 | F | Haemodialysis nurse | 10-20 | 20-30 | Daily |
| B4 | F | Healthcare assistant in haemodialysis unit | 1-2 | 2-3 | Most days |
| B5 | F | Consultant | 10-20 | 20-30 | Daily |
| B6 | F | Haemodialysis unit nurse manager | 30+ | 30+ | Most days |
| B7 | M | Consultant | 10-20 | 20-30 | Daily |
| B8 | F | Renal dietician | 10-20 | 30+ | Daily |
| B9 | F | Renal occupational therapist | 10-20 | 20-30 | Daily |
| B10 | M | Consultant | 20-30 | 30+ | Most days |
| B11 | F | Renal social worker | 10-20 | 20-30 | Daily |
| B12 | F | Specialist renal nurse | 5-10 | 10-20 | Daily |
| B13 | F | Specialist renal nurse | 10-20 | 20-30 | Weekly |
| B14 | F | Haemodialysis nurse | 20-30 | 30+ | 3-4 days per week |
| B15 | F | Specialist renal nurse | 10-20 | 20-30 | Weekly |
| B16 | F | Renal ward nurse | 10-20 | 30+ | Daily |
| A1 and B4 approached but not interviewed | | | | | |
